# Cloak Scavenges the Reactive Oxygen Species around the Larvae of *Drino inconspicuoides* (Diptera: Tachinidae)

**DOI:** 10.3390/insects14070602

**Published:** 2023-07-03

**Authors:** Kai Zhang, Satoshi Nakamura, Seiichi Furukawa

**Affiliations:** 1 Doctoral Program in Biosphere Resource Science and Technology, University of Tsukuba, Tsukuba 305-8572, Japan; zhangkai327@yahoo.co.jp; 2 Japan International Research Centre for Agricultural Sciences (JIRCAS), Tsukuba 305-8686, Japan; 0301tatsunakamura@gmail.com; 3 Institute of Life and Environmental Sciences, University of Tsukuba, Tsukuba 305-8572, Japan

**Keywords:** antioxidant, funnel, fat body, melanization, tachinid fly

## Abstract

**Simple Summary:**

The tachinid fly *Drino inconspicuoides* parasitizes various lepidopteran larvae, including Noctuidae and Nymphalidae. The adult female oviposits the eggs on the surface of the host, and the hatched larvae immediately penetrate into the host hemocoel and develop there during the larval stage. The caterpillar possesses immune defense mechanisms to eliminate such invaders, but the tachinid larva can avoid these defenses. Within 24 h, the parasitizing larva is surrounded by a black pigmented structure called the “funnel”, and the larva and the funnel are wrapped with a cottony structure called the “cloak”. The funnel is a physically rigid structure that penetrates the epidermis of the host, allowing the larva to breathe through contact with the outside air. This study aimed to clarify the function of the cloak, which has not previously been elucidated. Our findings suggest that formation of the funnel generates ROS, but that these are detoxified in the cloak to enhance the survival of the tachinid larva. The funnel and cloak are composed of host cells. Our study provides insight into the parasitic strategy of a tachinid fly by through which it ingeniously diverts host cells.

**Abstract:**

*Drino inconspicuoides* (Diptera: Tachinidae) is an endoparasitoid that develops inside the lepidopteran host. When the larva of *D. inconspicuoides* penetrates into the host, *Mythimna separata* (Lepidoptera: Noctuidae), the larva creates a cap-like structure, called the funnel, by using host hemocytes, forming a respiratory attachment to permit efficient respiration. A newly described cloudy and cottony structure, called the “cloak”, is formed outside the funnel within 24 h of parasitism. The cloak contains the host fat body and hemocytes. In this study, we aimed to clarify the function of the cloak, which has to date remained unknown. We hypothesized that the funnel generates reactive oxygen species (ROS) through melanization, and that the cloak detoxifies them. We confirmed that the black pigments of the funnel were caused by melanization, which inevitably generates ROS that are potentially harmful to the *D. inconspicuoides* larva inside the funnel. The cloak showed high activities of antioxidant enzymes, including superoxide dismutase, glutathione peroxidase, and catalase. These results suggest that the cloak scavenged the ROS from the melanized funnel through the diversion of antioxidant enzymes in the fat body, thereby protecting the *D. inconspicuoides* larva from oxidative damage.

## 1. Introduction

The Tachinidae are one of the largest groups of dipteran insects, with over 8500 species worldwide [1]. The tachinid fly species are a group of koinobiont parasitoids that allow their host to continue eating food and grow until finally killing it [2,3,4]. Since tachinid flies can utilize a variety of economically important insect pests of crops and forest as hosts, they are considered important biological control agents.

The tachinid fly *Drino inconspicuoides* (Baranov) parasitizes various lepidopteran insects such as *Polygonia caureum* (Linnaeus) (Lepidoptera: Nymphalidae), *Crocidolomia pavonana* (Fabricius) (Lepidoptera: Crambidae), *Hyphantria cunea* (Drury) (Lepidoptera: Arctiidae), *Helicoverpa armigera* (Hübner), *Mythimna separata* (Walker) (Lepidoptera: Noctuidae), and *Operophtera brumata* (Linnaeus) (Lepidoptera: Geometridae) [5,6,7,8]. We previously established an effective rearing method for *D. inconspicuoides* [9]. As many tachinid flies tend to be difficult to rear in the laboratory, *D. inconspicuoides* is a valuable model for studying parasitoid–host interactions.

*D. inconspicuoides* is a gregarious parasitoid; from two to three up to a dozen or more larvae can develop in a single host [8]. It is an ovolarviparous parasitoid whose embryo develops into the first instar within the egg while still in the mother’s oviduct [10]. After the female lays the incubated egg on the surface of the body of the host, the larva hatches within 1 min and rapidly penetrates the hemocoel of the host, passes through the host epidermis, anchors its bodies beneath the epidermis, and acquires nutrition from hemocytes and the fat body around it. Similar to other tachinid flies, *D. inconspicuoides* has three larval instars, and the period spent inside the host is approximately eight days [9]. When the *D. inconspicuoides* larva reaches the third instar, it moves freely in the host hemocoel and feeds on the tissues. It eventually kills the host, exits from the remains, and pupates. The pupal period averages 10 days [9].

Endoparasitoids that grow in the host’s hemocoel must evade the host’s immune system to survive. All insects have immune defense mechanisms that consist of cellular and humoral immune responses [11]. Cellular immune responses, including encapsulation, phagocytosis, and nodulation, are mainly mediated by hemocytes [12,13]. In lepidopteran insects, hemocytes are mainly classified into five types—prohemocytes, plasmatocytes, granulocytes, oenocytoids, and spherule cells [12]. Encapsulation is activated against larger invaders, such as parasitoids, and hemocytes surround the invaders to form hemocytic capsules that kill by suffocation [14,15]. Upon recognition of invaders, granulocytes (an adherent hemocyte type) attach first and form a single layer around the invader. Then, another adherent hemocyte, the plasmatocyte, covers the single layer to form a multi-layered capsule. Finally, granulocytes attach again to the outermost layer and undergo apoptosis, which may be a signal of the completion of capsule formation. Once the invader is entirely enclosed, the inner layer of the capsule is strongly melanized and shows deposits of black pigments [16,17].

In the melanization process, phenoloxidase (PO) is a critical enzyme; it exists in the hemolymph and hemocytes in the form of the precursor prophenoloxidase (proPO) [18]. In response to the intrusion of a foreign substance into the hemocoel, proPO is cleaved into active PO via a serine protease cascade [16]. PO first catalyzes the conversion of tyrosine to 3,4-dihydroxyphenylalanine (DOPA). Melanin with black pigmentation is finally synthesized via several reactions, including oxidation of quinoids mediated by PO [17,19]. During this process, various reactive oxygen species (ROS), such as superoxide anions (O_2_^•−^) and hydrogen peroxide (H_2_O_2_), are generated [20,21]. Since ROS are toxic, cause an uncontrolled increase in lipid peroxidation, and damage DNA and protein molecules [22,23], they play a vital role in insect immunity. In addition to suffocation, the invader in the hemocytic capsule is eliminated by ROS damage [24,25].

**Figure 1:**
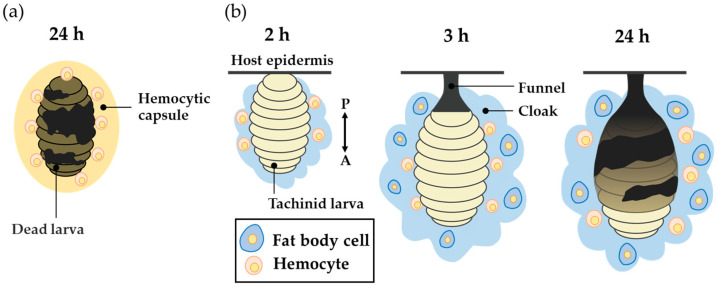
A schematic view of the encapsulation of transplanted freeze-killed *Drino inconspicuoides* larva and the early stage of its parasitization in the host, *Mythimna separata*. (**a**) The hemocytic capsule formed by encapsulation around freeze-killed *D. inconspicuoides* larva after 24 h of parasitism. (**b**) The process of cloak formation around the larva; diagrams from left to right show cloak status at 2, 3, and after 24 h of parasitism. The first-instar *D. inconspicuoides* is first enveloped by the host hemocytes, after which the fat body of the host covers the hemocyte layer; finally, the two tissues are mixed. The double-headed arrow indicates the anterior–posterior (A/P) axis of the larva.

However, the larva of a tachinid fly that successfully parasitizes a host is not enclosed by the hemocytic capsule formed by encapsulation. Instead, a cap-like structure called the “funnel”, which wraps the posterior part of the larva, is formed [26,27,28,29]. The funnel allows the tachinid larva to have direct contact with the outside air via the body wall of the host, thereby preventing death by suffocation. When a freeze-killed *D. inconspicuoides* larva was experimentally transplanted into a host, the oriental armyworm *M. separata*, it became surrounded by the hemocytic capsule and melanized by encapsulation [30] (Figure 1a), suggesting that the parasitizing live tachinid larval parasitoids actively form the funnel. The funnel consists of host hemocytes [28,29], which suggests that the funnel is formed in response to active manipulation of the host immune system by parasitoids. The tachinid larva utilizes the host hemocytes, whose original function is to kill the parasitoid via encapsulation to generate a respiratory apparatus. Salt [27] interpreted this strategy of parasitoids as resistance by deflection of the host’s effort.

We previously observed a unique cloudy and cottony structure in connection with this process, which we named the “cloak” [30]. The cloak is not formed around dead *D. inconspicuoides* larvae or microbeads, but envelops the parasitizing live larva of *D. inconspicuoides* and its surrounding funnel in a parasitized host, *M. separata* (Figure 1b). We have clarified the following by employing a combination of techniques using genetic markers. At after 2 h of parasitism, the *D. inconspicuoides* larva is surrounded by a cloudy material that contains only the host hemocytes. After 3 h, the cottony mass containing the host fat body and hemocytes gradually accumulates around the tachinid larva. The formation of this cloak derived from host materials is completed within 24 h [30]. The purpose of the present study was to clarify the function of the cloak, which has to date remained uninvestigated. We hypothesized that the funnel generates ROS through melanization, and that the cloak detoxifies them. We investigated ROS levels and the activities of antioxidant enzymes, including superoxide dismutase (SOD), glutathione peroxidase (GPx), and catalase in the funnel and cloak.

## 2. Materials and Methods

### 2.1. Insect Rearing

We used a colony of *D. inconspicuoides* originating from parasitized larvae of the fall webworm *Hyphantria cunea* (Drury) (Lepidoptera: Erebidae), which were collected in Tsukuba, Japan (36°03′ N, 140°05′ E), in September and October 2010. *Mythimna separata* was supplied from stock cultures stored at Takeda Chemical Industries, Ltd. (Osaka, Japan) [31], and maintained in the Laboratory of Applied Entomology and Zoology, University of Tsukuba, Japan. The parasitoid flies were reared according to the method described by Kalyebi and Nakamura [9], using larvae of *M. separata* as host insects at 25 °C under a 16 h light: 8 h dark photoperiod. The adult flies were maintained with water and sugar cubes at 20 °C, 60–70% relative humidity, and a 16 h light:8 h dark photoperiod. The larvae of *M. separata* were fed an artificial diet (Silkmate; Nosan Corporation, Yokohama, Japan).

### 2.2. Sample Collection

*Drino inconspicuoides* typically lays multiple eggs on a single caterpillar host. In this experiment, we placed a mated female of *D. inconspicuoides* in a 9 cm diameter petri dish along with a sixth-instar larva of *M. separata*, and she was removed after she laid one egg on the caterpillar larvae. After 24 h of parasitism, the hosts were dissected under an MZ12 stereoscopic microscope (Leica Microsystems, Wetzlar, Germany). The tachinid larva, funnel, and cloak were taken together out of the hosts, the tachinid larva was removed out of the funnel, and the cloak was peeled off from the funnel. For further purification of the funnel, it was washed multiple times with phosphate-buffered saline (PBS), and centrifuged to remove any residual cloak components. Hemocytes and the fat body samples were also prepared from the five parasitized hosts from which tachinid larvae were sampled. To collect host cells that form hemocytic capsule formed by encapsulation, a hemocytic capsule was artificially prepared by transplanting micro-polystyrene beads (Φ600 μm; Polysciences, PA, USA) into ice-anesthetized sixth-instar larvae of *M. separata*. The beads were transplanted into an abdominal leg and the leg was immediately tied with a thread to prevent hemolymph leakage. The microbeads were left in the host hemocoel for 24 h to ensure the full melanization of the artificial hemocytic capsules formed around the beads, and were surgically extracted from the larvae. All the samples were stored at −80 °C until being subjected to PO activity, ROS measurement, and antioxidant enzyme assays.

### 2.3. Measurement of PO Activity

PO activity was measured following the method described by Laughton and Siva-Jothy [32]. The samples were homogenized in 40 µL PBS and centrifuged at 6000× *g* for 1 min. The supernatant (20 μL) was mixed with 20 µL of 5.75 mM L-DOPA (Sigma-Aldrich, St. Louis, MO, USA) and then diluted with 960 µL distilled water. After 20 min, the optical absorbance at 490 nm was read in a plate reader (Biochrom, Cambridge, UK). One unit of PO activity was defined as ΔA490 = 0.001 after 10 min of incubation. PO activity was normalized by the amount of total proteins measured using the Qubit Protein Assay Kit (Thermo Fisher Scientific, Waltham, MA, USA) and a Quantus Fluorometer (Promega, Madison, WI, USA).

### 2.4. Measurement of ROS Content

ROS levels were determined using the OxiSelect InVitro ROS/RNS Assay Kit Green Fluorescence (Cell Biolabs, San Diego, CA, USA) according to the manufacturer’s instructions. ROS in the samples react with dichlorodihydrofluorescin (DCFH), rapidly oxidizing to the highly fluorescent 2′, 7′-dichlorodihydrofluorescein (DCF). Each sample was homogenized in 40 µL PBS on ice, centrifuged at 10,000× *g* for 5 min, and the supernatant was allowed to react with the provided DCFH probe for 45 min at 25 °C. Oxidation levels were measured from the fluorescence emission at 530 nm when excited at 480 nm using a microplate reader (Multiskan Ascent, Thermo Fisher Scientific). The amount of ROS was normalized by the amount of total proteins and expressed in terms of nmol of DCF per mg of protein.

### 2.5. Antioxidant Enzyme Assay

The activities of three antioxidant enzymes, SOD, GPx, and catalase, were analyzed. SOD activity was measured using the Colorimetric EpiQuik Superoxide Dismutase Activity/Inhibition Assay Kit (EpiGentek, Farmingdale, NY, USA). The principle of this assay is based on the formation of a colored water-soluble formazan upon reduction of a dye with superoxide anions. The samples were incubated with a water-soluble tetrazolium salt, and the optical absorbance at 470 nm was measured. The amount of SOD required to inhibit the rate of reduction of cytochrome c by 50% was defined as 1 unit of activity. GPx activity was detected using the Amplite Fluorimetric Glutathione Peroxidase Assay Kit (AAT Bioquest, Sunnyvale, CA, USA). This assay is based on the oxidation of glutathione (GSH) to oxidized glutathione (GSSG) catalyzed by glutathione peroxidase. Each sample was incubated with an NADP probe; then, the fluorescence emission at 480 nm when excited at 420 nm was measured. One unit of GPx was defined as the amount of enzyme capable of oxidizing 1.0 µmol GSH to GSSG per min. Catalase activity was measured using the DetectX Catalase Fluorescent Assay Kit (Arbor Assays, Ann Arbor, MI, USA). The principle of this assay is based on the reaction of horseradish peroxidase (HRP) with the substrate in the presence of hydrogen peroxide to convert the colorless substrate into a fluorescent product. Each sample was mixed with HRP, and the fluorescence emission at 590 nm when excited at 520 nm was measured. One unit of catalase activity was defined as the amount of enzyme that decomposed 1.0 µmol H_2_O_2_ per min. All activities of the studied antioxidative enzymes were normalized by the amount of total proteins.

### 2.6. Statistical Analysis

Statistically significant differences between groups were assessed using a one-way analysis of variance (ANOVA) followed by the Tukey–Kramer multiple comparison test. All analyses were conducted using the R software (version 4.0.3) [33]. *p*-values < 0.01 were considered statistically significant.

## 3. Results

### 3.1. Melanization of the Funnel around D. inconspicuoides Larva

**Figure 2:**
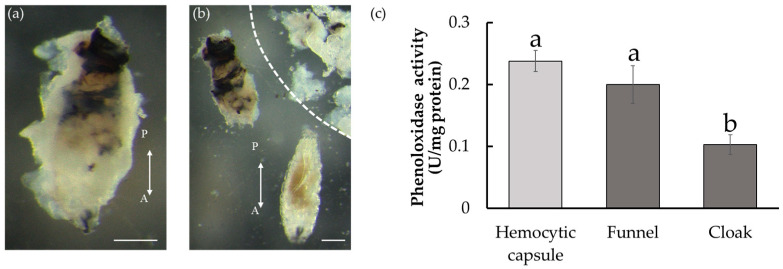
Host-derived structures formed around *Drino inconspicuoides* larva developing in *Mythimina separata* caterpillars and phenoloxidase activity. (**a**) Larva after 24 h of parasitism. The parasitized larva is encased in the white cottony cloak. (**b**) Larva (bottom), the funnel (upper left), and the cloak peeled off from the funnel (upper right; zoned by a dotted line). The funnel under the cloak was rigid with black pigmentation, particularly in the posterior region. Scale bars = 0.25 mm. Double-headed arrows indicate the anterior–posterior (A/P) axes of the larvae. (**c**) Phenoloxidase activity of the hemocytic capsule, the funnel, and the cloak. Each value is expressed as the mean ± standard deviation of samples (*n* = 5). Different letters above each bar denote significant differences (*p* < 0.01).

The *D. inconspicuoides* larva in the host is surrounded by host material. The funnel adhering to the larva features black pigmentations, whereas the cloak does not. The pigmentation of the funnel is thought to be due to melanization, similar to that in the hemocytic capsule formed by encapsulation, but this has not been experimentally demonstrated. To confirm that the black pigmentation on the funnel is derived from melanin, we measured the PO activity that mediates melanization. After removing the tachinid larvae and structures surrounding them from the host after 24 h of parasitism, we isolated the blackened funnel and the cottony cloak from the *D. inconspicuoides* larvae (Figure 2a,b). The funnel showed a significantly higher PO activity than the cloak (ANOVA, F_2,12_ = 43.5, *p* < 0.01; Tukey–Kramer’s test, Figure 2c). To obtain a hemocytic capsule consisting of only host materials, we prepared artificial capsules by transplanting microbeads into *M. separata*. We used a microbead instead of a freeze-killed *D. inconspicuoides* larva, as separating the dead larva and host-derived parts was not feasible. The activity of the funnel was comparable to that of the artificial capsule that imitated the hemocytic capsule intended to kill the parasitoid. These observations suggest that the funnel is melanized and potentially harmful to the *D. inconspicuoides* larva.

### 3.2. Production of ROS in the Funnel around D. inconspicuoides Larvae

**Figure 3:**
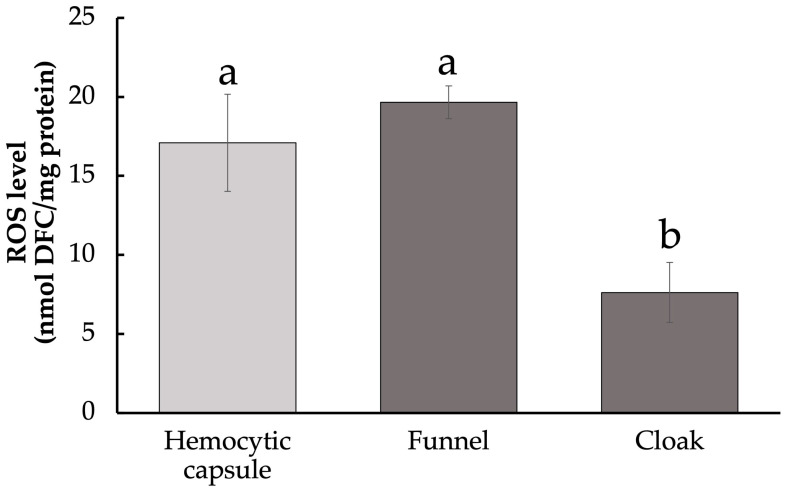
Levels of reactive oxygen species in host-derived structures surrounding developing *Drino inconspicuoides* larva. Values are expressed as mean ± standard deviation (Hemocytic capsule, *n* = 5; Funnel, Cloak, *n* = 10). Different letters above each bar denote significant differences (*p* < 0.01).

Since melanization inevitably causes the production of ROS, which can be a host defensive response to cause damage to the parasitoid, we measured the levels of ROS in the funnel and cloak. ROS include O_2_^•−^, ·OH, and H_2_O_2_, which were quantified collectively. ROS content in the melanized funnel was as high as in the artificial hemocytic capsule (ANOVA, F_2,22_ = 66.89, *p* < 0.01; Tukey–Kramer’s test, Figure 3). In contrast, ROS levels in the cloak were significantly lower than those in the funnel. The low ROS levels of the cloak may be explained by low PO activity, but it is also possible that the cloak exhibits antioxidant effects of actively removing ROS.

### 3.3. Activities of Three Antioxidant Enzymes in the Cloak

In animals, ROS are scavenged by many enzymatic antioxidants, such as SOD, GPx, catalase, and glutathione-S-transferase, and some non-enzymatic antioxidants, including ascorbic acid, thiols, and 𝛼-tocopherols [34,35]. We examined the activities of three antioxidant enzymes in the funnel and cloak to examine whether the cloak actively removes ROS.

**Figure 4:**
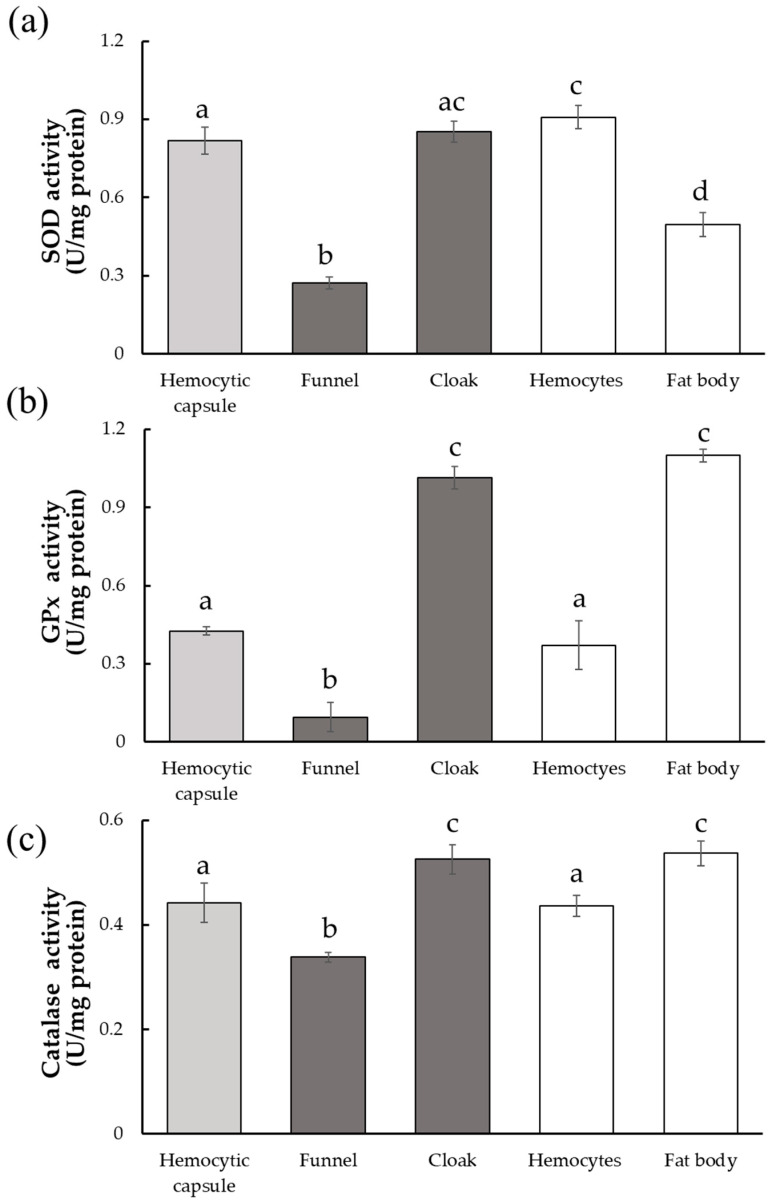
Activities of three antioxidant enzymes in host-derived structures surrounding developing *Drino inconspicuoides* larva. (**a**) Superoxidase dismutase (SOD) activity; (**b**) Glutathione peroxidase (GPx) activity; (**c**) Catalase activity. Each value is expressed as the mean ± standard deviation of samples (*n* = 5). Different letters above each bar denote significant differences (*p* < 0.01).

O_2_^•−^ is the first ROS molecule generated during the melanization process and converted into H_2_O_2_ by SOD in an early step of the ROS detoxification pathway. SOD activity of the cloak was higher than that of the funnel and comparable to that of the hemocytic capsule (ANOVA, F_4,20_ = 215.4, *p* < 0.01; Tukey–Kramer’s test, Figure 4a), suggesting that conversion of O_2_^•−^ to H_2_O_2_ occurred mainly in the cloak rather than in the funnel. The hemocytic capsule formed by encapsulation and the respiratory funnel are derived from hemocytes, whereas the cloak contains fat body tissue in addition to hemocyte-derived structures. We, therefore, also measured the SOD activities of hemocytes and the fat body isolated from *M. separata* larvae. SOD activity was significantly higher in hemocytes than in the fat body, suggesting that the high activity in the cloak is associated with the contained hemocytes.

GPx and catalase are enzymes that act independently on H_2_O_2_, but both of them convert H_2_O_2_ into non-toxic H_2_O. GPx activity was much higher in the cloak than in the funnel (ANOVA, F_4,20_ = 293.2, *p* < 0.01; F_4,20_ = 58.47, *p* < 0.01; Tukey–Kramer’s test, Figure 4b,c). Upon comparing the host tissues, the activity in the fat body was higher than that in the hemocytes, suggesting that the high GPx activity in the cloak is mainly due to the presence of the host fat body. Catalase activity also showed a trend similar to that of GPx (ANOVA, F_4,20_ = 293.2, *p* < 0.01; Tukey–Kramer’s test, Figure 4c), although the differences in activity between the tissues were less than those observed for GPx activity.

## 4. Discussion

Parasitoid larvae developing inside hosts that lack countermeasures against host immune defenses are typically surrounded by hemocytic capsules formed by encapsulation and are suffocated. In addition, the ROS generated during melanization inside the hemocytic capsule are thought to contribute to the elimination of parasitoids. The artificial hemocytic capsule we prepared in this study had high PO activity. They contained high levels of ROS (Figure 2c and Figure 3), indicating that the ROS generated during melanization accumulate within the capsules and cause damage to the invaders. In this study, we first measured the PO activity in the funnel and the findings indicate that its black pigmentation was derived from melanization, as was that of the hemocytic capsule (Figure 2c). Melanization inevitably leads to the production of ROS, which are harmful to the tachinid larva in the funnel. We postulated that the cloak formed around the funnel detoxified these ROS.

**Figure 5:**
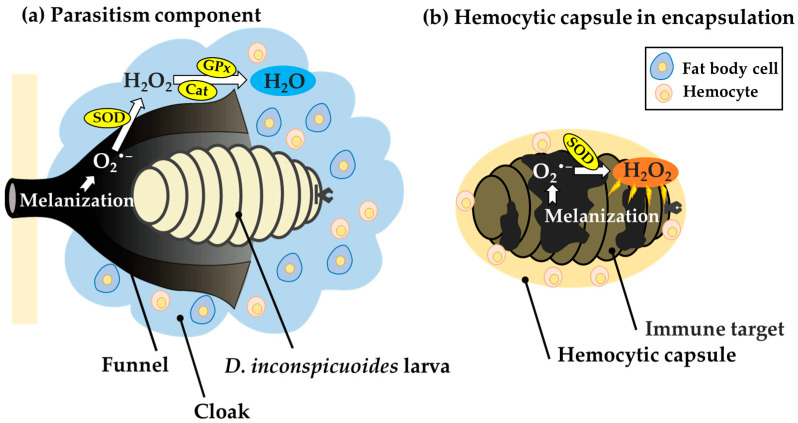
Comparison of putative metabolic pathways of reactive oxygen species (ROS) in host-derived structures surrounding developing *Drino inconspicuoides* larvae and hemocytic capsules during encapsulation. (**a**) In the structure formed around the *D. inconspicuoides* larva, O_2_^•−^, a type of ROS, is generated from the melanization of the funnel. The O_2_^•−^ is detoxified to H_2_O via H_2_O_2_ owing to the high SOD, GPx, and catalase activities of the cloak containing the host fat body, allowing the tachinid larva to avoid damage from the ROS. (**b**) In the hemolytic capsule in encapsulation, melanization activated around invaders leads to the production of O_2_^•−^, which is converted into highly toxic H_2_O_2_ by SOD. However, H_2_O_2_ is not detoxified into H_2_O due to low activities of GPx and catalase in the capsules and thus causes damage to invaders as a toxic molecule. SOD: superoxide dismutase; GPx: Glutathione peroxidase; Cat: Catalase.

The first ROS produced during melanization is O_2_^•−^, which converted to H_2_O_2_ by SOD [36]. Figure 5a summarizes the putative mechanism of the detoxification of the ROS by the cloak. ROS levels were higher in the funnel than in the cloak (Figure 3), whereas SOD activity was lower in the funnel than in the cloak (Figure 4a), suggesting that the high ROS levels in the funnel are attributable mainly to O_2_^•−^. The high SOD activity in the cloak suggests that H_2_O_2_ is produced using the O_2_^•−^ in the funnel as the substrate. In the cloak, the activities of GPx and catalase, both of which convert H_2_O_2_ to non-toxic H_2_O [37,38], were higher than those in the funnel (Figure 4b,c), suggesting that the detoxification of O_2_^•−^ to H_2_O through H_2_O_2_ takes place in the cloak. ROS levels in the cloak were low (Figure 3), possibly because H_2_O_2_ is rapidly converted to H_2_O in the cloak. It is conceivable that melanization of the funnel produces O_2_^•−^, which is detoxified into H_2_O in the cloak. This prevents the O_2_^•−^ from the funnel from seriously damaging *D. inconspicuoides* larvae inside the funnel. Measurements of GPx and catalase activity (Figure 4b,c) suggest that GPx plays a more dominant role than catalase in detoxifying H_2_O_2_ in the cloak. On the other hand, the hemocytic capsule formed by encapsulation had high SOD activity but low GPx and catalase activities (Figure 4a–c); this indicates that the O_2_^•−^ generated during melanization around the invaders is converted to H_2_O_2_ but not detoxified to H_2_O as it is in the cloak. H_2_O_2_, which is more cytotoxic than O_2_^•−^ [39], is known to be a major cause of oxidative damage [40,41], and its elimination is critical to the survival of parasitoid wasps [23]. This highly toxic H_2_O_2_ in the hemocytic capsule acts to efficiently kill the invader in the capsule (Figure 5b). 

We previously examined the origin of the cells contained in the funnel and cloak by performing PCR using specific primer sets for the *M. separata* and *D. inconspicuoides* [30]. The result revealed that both structures exclusively contained host cells and no cells derived from the tachinid fly. In general, SOD, GPx, and catalase lack a signal peptide necessary for extracellular secretion [42,43,44,45,46]. This implies that the origin of the antioxidant enzymes detected in the funnel and cloak are derived from the host cells. They are synthesized by the host cells, and not secreted from the tachinid larva. 

The hemocytic capsule and funnel are made of the host’s hemocytes, but the SOD activity of the funnel was significantly lower than that of hemocytic capsule and hemocytes. This may suggest that SOD is downregulated in the hemocytes constituting the funnel. Reducing conversion of less toxic O_2_^•−^ into highly toxic H_2_O_2_ by suppressing the SOD activity in the funnel is beneficial for the tachinid larvae. It has been reported that parasitism of *E. bombycis* affects the activities of antioxidant enzymes, including SOD and catalase, in the silkworm *Bombyx mori* (Linnaeus) (Lepidoptera: Bombycidae) [47]. Tachinid larvae may also be capable of influencing enzyme expression in hemocytes of their hosts.

The formation of the cloak outside the funnel raises the question of who is inducing the cloak formation. One possibility is that the tachinid larva induces the cloak to efficiently release the O_2_^•−^ from the funnel. Another possibility is that the host forms the cloak as a defensive reaction to detoxify O_2_^•−^ that leaks out of the funnel as a consequence of the suppression of SOD activity by the tachinid larva. We recently discovered that the salivary gland of *D. inconspicuoides* larvae contains substances that attract cells of the host fat body (data not shown); we therefore suggest that it is more likely that the tachinid larva, rather than the host, induces cloak formation to efficiently detoxify ROS produced in the funnel.

The mechanisms by which parasitoids evade host immune defenses have been studied in greater detail in parasitic wasps than in tachinid flies. Most females of endoparasitoid wasps that deposit their eggs into the host inject immune-suppressive factors, such as polydnaviruses and venom, to suppress PO activity, production of ROS, and encapsulation [48,49,50,51,52]. In contrast, many species of tachinid flies cannot inject such immune-suppressive factors, requiring the hatched larvae to evade the host’s immune defenses on their own, for which they have developed various strategies. In *D. inconspicuoides* and *Exorista bombycis* (Louis) (Diptera: Tachinidae), the larvae secrete immune-suppressive factors [53,54]. As a strategy that is not found in parasitic wasps, some tachinid species migrate and develop in sites where host hemocytes cannot reach them, such as the salivary glands, ganglia, or midgut [55,56]. Creating a breathing funnel is also unique to tachinid flies. This strategy seems to be sophisticated in that the tachinid larvae utilize the host’s materials to protect themselves, making it difficult for the hosts to develop countermeasures with their own materials.

The high antioxidant enzyme activity in the cloak suggests that it contributes to the scavenging of toxic ROS from the funnel, which provide the *D. inconspicuoides* larvae a suitable environment to develop. The presence of cloak has only been reported in *D. inconspicuoides*, and it remains to be investigated whether cloak formation is also present in other parasitoids. Future studies on the molecular mechanisms of transformation of host tissues into funnels and cloaks in *D. inconspuoides*, and possibly in other parasitoids, will lead to a more comprehensive understanding of parasitic strategies of tachinid flies.

## Data Availability

The datasets and analysis protocols used during the current study are available from the corresponding author on request.
